# Synthesis and Anticancer Properties of Silver(I) Complexes Containing 2,6-Bis(substituted)pyridine Derivatives

**DOI:** 10.1155/2013/256836

**Published:** 2014-10-22

**Authors:** Korany A. Ali, Mokhles M. Abd-Elzaher, Khaled Mahmoud

**Affiliations:** ^1^Applied Organic Chemistry Department, National Research Centre, Dokki, Cairo 12622, Egypt; ^2^Inorganic Chemistry Department, National Research Centre, Dokki, Cairo 12622, Egypt; ^3^Department of Pharmacognosy, National Research Centre, Dokki, Cairo 12622, Egypt

## Abstract

Several new 2,6-bis(substituted)pyridine ligands and 2,6-bis(substituted)pyridine Ag(I) nitrate complexes were synthesized and characterized spectroscopically. The newly synthesized ligands include pyridine-2,6-bis(3-oxopropanenitrile) (**1**), pyridine-2,6-bis(2-cyano-*N*-phenyl-3-oxopropanethioamide) (**2**), and pyridine-2,6-bis((*E*)-2-(2-phenylhydrazono)-3-oxopropanenitrile) (**3**). The newly synthesized ligands and silver(I) complexes were evaluated for their *in vitro* anticancer activity against four human cancer cell lines including hepatocellular carcinoma (HePG2), lung adenocarcinoma (A549), colon carcinoma (HT29), and breast adenocarcinoma (MCF7). Most of the newly synthesized silver(I) complexes exhibited better activity than the ligands, and the results have been compared with doxorubicin as a reference drug.

## 1. Introduction

Chemical and biological activity of the organic complexes is not possessed only by the metal or organic ligand but also this activity can be fine-tuned by subtle changes in the electronic and steric properties of the complexes or by variation of the oxidation state of the metal. These features provide a versatile platform for drug design that is now being exploited in several areas. For centuries, silver compounds have been known to possess interesting biological properties that show potent antibacterial properties [1–4]. Also, they were popular remedies for tetanus and rheumatism in the 19th century and for colds and gonorrhea before the advent of antibiotics in the early part of the 20th century [5]. Additionally, silver compounds used for treating mental illness, epilepsy, and nicotine addiction [6, 7]. Furthermore, silver compounds have reemerged as a viable treatment option for infections encountered in burns, open wounds, and chronic ulcers [8–11]. On the other hand, functionalized pyridine derivatives are gaining a great deal of interest in medicinal and organic synthesis, where some of pyridine derivatives are used as bactericides [12], fungicides [13], and anticancer agents [14–17]. In view of these observations and in continuation of our current interest in the synthesis of organic compounds for biological evaluations [18–25] and our interest in the chemistry of 2,6-disubstituted pyridine derivatives [26–29], we described herein a facile synthesis of novel silver complex with some of the newly synthesized 2,6-disubstituted pyridine ligands. The newly synthesized compounds were evaluated for their* in vitro* anticancer activity against four human cancer cell lines including hepatocellular carcinoma (HePG2), lung adenocarcinoma (A549), colon carcinoma (HT29), and breast adenocarcinoma (MCF7).

## 2. Results and Discussion

### 2.1. Synthesis and Characterization of 2,6-bis(substituted)pyridine Ligands

Three ligands have been prepared, pyridine-2,6-bis(3-oxopropanenitrile) (**1**), pyridine-2,6-bis(2-cyano-*N*-phenyl-3-oxopropanethioamide) (**2**), and pyridine-2,6-bis((*E*)-2-(2-phenylhydrazono)-3-oxopropanenitrile) (**3**). Structures of the synthesized ligands were confirmed on the basis of their elemental analysis and spectral data. The elemental analyses of the prepared ligands are consistent with the calculated results from the empirical formula of each one. Ligand** 1** was prepared using ethyl 2,6-pyridine dicarboxylate and acetonitrile in dry THF and in presence of sodium hydride [26]. The IR spectrum of ligand** 1** revealed two bands at 2264 and 1720 cm^−1^ which were assigned to CN and C=O groups, respectively. The band that appeared at 1566 cm^−1^ was assigned to C=N of the pyridine moiety. ^1^H NMR of** 1** was carried out using DMSO-*d*_6_ as a solvent. The signal of the two CH_2_ groups was appeared at 3.74 ppm, whereas the signals of the pyridine moiety were appeared at 8.05–8.31 ppm.

**Scheme 1:**
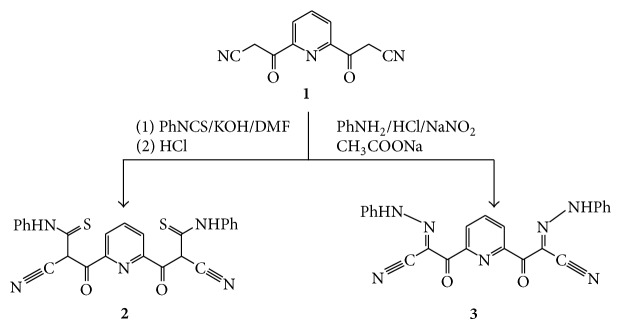
Synthetic pathway for the formation of ligands** 2** and** 3**.

To increase solubility and hydrogen bonds capabilities of the synthesized ligands we have used pyridine-2,6-bis(3-oxopropanenitrile) (**1**) as starting material for the preparation of Ligand** 2** and Ligand** 3**. Ligand** 2** was prepared as brown powder in a good yield using pyridine-2,6-bis(3-oxopropanenitrile) (**1**) and phenyl isothiocyanate in DMF in the presence of KOH with stirring for 6 h (Scheme 1).

Four essential bands were found in the IR spectra of Ligand** 2** at 3191, 2210, 1655, and 1555 cm^−1^. These bands were assigned to (NH), (CN), (C=O), and (C=N in pyridine), respectively. ^1^H NMR was carried out using DMSO-*d*_6_ at room temperature. Signal of the two CH group was appeared at 3.60 ppm, whereas the aromatic and the pyridine protons were appeared in the expected region at 7.25–7.43 and 7.74–8.02 ppm, respectively, in addition to singlet signal at 12.02 ppm corresponding to 2NH protons. Elemental analysis, ^13^C NMR, and also mass spectra of** 2** were consistent with the proposed structure.

Compound** 1** was coupled with benzene diazonium salt to yield coupling product pyridine-2,6-bis((*E*)-2-(2-phenylhydrazono)-3-oxopropanenitrile) (**3**) (Scheme 1).

Characterization of ligand** 3** was confirmed on the basis of its elemental analysis and spectral data. For example, four bands in IR spectrum at 3187, 2213, 1674, and 1549 cm^−1^ correspond to NH, CN, C=O, and C=N group, respectively. Its ^1^H NMR spectrum revealed signals at the range 7.10–7.28 ppm corresponding to the phenyl protons and multiplet at 7.97–8.20 ppm for pyridine protons in addition to singlet signal at 12.63 ppm corresponding to 2NH protons. Mass spectrum and elemental analysis confirmed also the structure of** 3**. All the ligands were isolated in a good yield, and they are stable in air and light.

### 2.2. Synthesis and Characterization of 2,6-bis(substituted)pyridine Ag(I) Nitrate Complexes

**Scheme 2:**
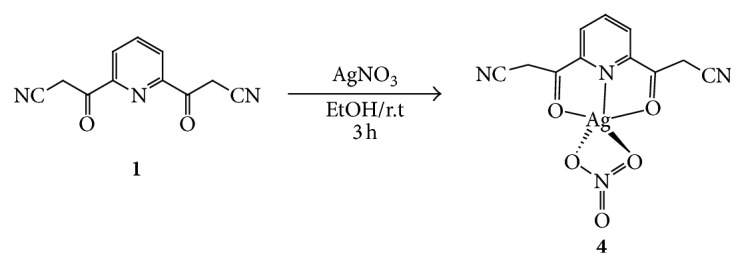
Synthetic pathway for the formation of complex** 4**.

In general, all of the ligands have good ability to form complexes due to the presence of several donor atoms in each ligand. So, preparations of the complexes were carried out easily and gave good yields. Formations of the complexes were observed from the change in colour of the ligand in the mixture as well as from the precipitation of the complexes (in the most cases). The silver complex** 4** was prepared as yellow solid in good yield from the reaction of ligand** 1** with silver nitrate (1 : 1 molar ratio), in ethanol, at room temperature (Scheme 2).

**Scheme 3:**
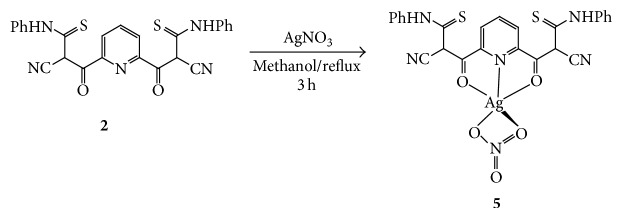
Synthetic pathway for the formation of complex** 5**.

The silver complex** 5** was prepared using ligand** 2** with silver nitrate (1 : 1 molar ratio), in methanol, under reflux condition (Scheme 3). The yield was good, and the colour of the prepared complex was brown.

**Scheme 4:**
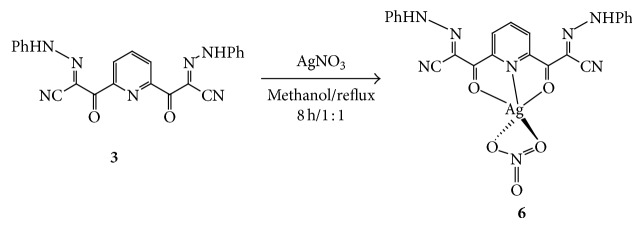
Synthetic pathway for the formation of complex** 6**.

Refluxing of ligand** 3** with AgNO_3_ in methanol affords yellow precipitate of complex** 6** in good yield (Scheme 4).

Characterization of the synthesized complexes was carried out using IR, NMR, and elemental analysis. All the complexes are stable in air and light.

The elemental analyses data of the complexes are consistent with the calculated results from the empirical formula of each compound. The IR spectra of the metal complexes were recorded in KBr.

It was found that C=O frequency of each of the complexes (**4**–**6**) was shifted to lower values compared with that of the ligands (in the range 1625–1609 cm^−1^) indicating the participation of the C=O group in a coordinated bond with the metal ions. Confirmation of this coordinated bond was observed in the IR spectra; it was found that a new band in the complexes appeared at 460–561 cm^−1^; this band was not found in the free ligand and was assigned to C=O–Ag bond. Also, the band at approx. 1565 cm^−1^ which was assigned to the frequency of the C=N in the pyridine moiety was slightly shifted to lower frequencies (11–52 cm^−1^) in the spectra of the metal complexes. This shift suggests that the coordination takes place also through the pyridine nitrogen to the metal ion. These results were confirmed also from the presence of a new band at about 440 cm^−1^ in the complexes which was assigned to the Ag–N bond. This band was observed only in the spectra of the metal complexes and not in the spectra of the ligands, thus confirming the participation of the nitrogen atom in the formed complex [30–33].

The ^1^H NMR spectra of the complexes** 4**–**6** were recorded at room temperature using DMSO-*d*_6_ as a solvent. The spectra of the complexes showed nearly the same signals appeared in the spectra of ligands with slightly downfield shift, which may be due to complex formation of the C=O and the pyridinyl nitrogen with metal ions.

### 2.3. *In Vitro* Anticancer Screening

*In vitro* anticancer activity evaluation of the newly synthesized compounds was carried out against four human cancer cell lines including hepatocellular carcinoma (HePG2), lung adenocarcinoma (A549), colon carcinoma (HT29), and breast adenocarcinoma (MCF7) using MTT method [34]. Doxorubicin HCl is one of the most effective anticancer agents was used as a reference drug in this study. The relationship between drug concentrations and cell viability was plotted to calculate IC_50_ (*μ*M) the value which corresponds to the concentration required for 50% inhibition of cell viability.  Table 1 shows the* in vitro *cytotoxic activity of the newly synthesized compounds against the tested cancer cell lines; where some compounds revealed significant activity compared to doxorubicin. From Table 1, it was observed that most of the synthesized ligands showed low to moderate activity, while most of the prepared silver complexes exhibit excellent cytotoxic activity against tested cancer cell lines.

In HePG2 silver complexes** 4** (IC_50_ = 1.52 *μ*M) and** 6** (IC_50_ = 1.62 *μ*M) revealed significant activity compared to doxorubicin (IC_50_ = 0.4.61 *μ*M). In A549 silver complexes** 4** (IC_50_ = 1.41 *μ*M),** 5** (IC_50_ = 1.08 *μ*M), and** 6** (IC_50_ = 3.71 *μ*M) revealed significant activity compared to doxorubicin (IC_50_ = 3.51 *μ*M). In HT29 silver complex** 4** (IC_50_ = 1.75 *μ*M) revealed significant activity compared to doxorubicin (IC_50_ = 5.22 *μ*M). In MCF7 silver complexes** 4** (IC_50_ = 3.67 *μ*M) and** 6** (IC_50_ = 3.66 *μ*M) revealed moderate activity compared to doxorubicin (IC_50_ = 2.78 *μ*M).

## 3. Conclusions

In the present study we have described the synthesis and characterizations of new silver complexes of 2,6-disubstituted pyridine ligands. The synthesized ligands and complexes were tested with doxorubicin on four human cancer cell lines including hepatocellular carcinoma (HePG2), lung adenocarcinoma (A549), colon carcinoma (HT29), and breast adenocarcinoma (MCF7) using MTT method. The entire synthesized complex displayed significant activity more than the corresponding ligands. While most of the prepared silver complexes exhibit excellent cytotoxic activity against tested cancer cell lines more than the reference drug. In the near future we will conduct some studies related to the mechanism of actions of the synthesized Ag(I) complexes, for example, assays against potential targets, particularly DNA: DNA binding properties of the proposed complexes by viscosity measurements, electronic absorption, fluorescence, and/or DNA electrophoresis.

## 4. Experimental

### 4.1. Chemistry

*General: *all melting points were measured on a Gallenkamp melting point apparatus. The infrared spectra were recorded in potassium bromide discs on a Pye Unicam SP 3-300 and Shimadzu FT IR 8101 PC infrared spectrophotometers. The NMR spectra were recorded on a Varian Mercury VXR-300 NMR spectrometer. ^1^H NMR (300 MHz) and ^13^C NMR (75.46 MHz) were run in deuterated dimethyl sulfoxide (DMSO-*d*_6_). Chemical shifts were related to that of the solvent. Mass spectra (EI, 70 ev) were recorded on a Shimadzu GCMS-QP1000 mass spectrometer. Elemental analyses were carried out at the Microanalytical Centre of Cairo University, Giza, Egypt, and recorded on Elementar-Vario EL (Germany) automatic analyzer. All reactions were followed by TLC (Silica gel, Aluminium Sheets 60 F_254_, Merck).

### 4.2. Preparation of 2,6-bis(substituted)pyridine Ligands

#### 4.2.1. Pyridine-2,6-bis(3-oxopropanenitrile) (**1**)

To a mixture of ethyl pyridine-2,6-dicarboxylate (4.45 g, 20 mmol) and acetonitrile (2.70 mL, 50 mmol), in dry THF (5 mL), sodium hydride (2 g, 50%) was added. The reaction mixture was refluxed for 2 h and then allowed to cool. The precipitated product was filtered off, washed with diethyl ether, and dried. The crude product was dissolved in water (30 mL), and the resulting alkaline solution was treated with concentrated hydrochloric acid until it becomes slightly acidic (pH 5). The precipitated solid product was filtered off, washed with water, dried, and finally recrystallized from MeOH to afford brown crystals of pyridine-2,6-bis(3-oxopropanenitrile) (**1**) [26]. Yield (3.41 g, 80%); mp: 185-186°C; IR (KBr pellet, cm^−1^): 2264, 1720, 1566, 1339, 1206, 1024, 927, 835. ^1^H NMR (300 MHz, DMSO-*d*_6_): *δ* 3.74 (s, 4H, 2CH_2_), 8.05–8.31 (m, 3H, pyridine). ^13^C NMR (75 MHz, DMSO-*d*_6_): *δ* 29.6, 116.4, 123.7, 126.4, 165.7, 190.7. MS (*m/z, *%): 213 [M^+^] (75), 145 (100). Anal. Calcd. for C_11_H_7_N_3_O_2_ (213.19): C, 61.97; H, 3.31; N, 19.71. Found: C, 61.94; H, 3.34; N, 19.75.

#### 4.2.2. Pyridine-2,6-bis(2-cyano-N-phenyl-3-oxopropanethioamide) (**2**)

To a stirred solution of potassium hydroxide (1.12 g, 20 mmol) in DMF (20 mL) pyridine-2,6-bis(3-oxopropanenitrile) (**1**) (2.13 g, 10 mmol) was added. The mixture was stirred for 30 min, and then phenyl isothiocyanate (2.70 g, 20 mmol) was added. Stirring was continued for 6 h, and then the mixture was poured over crushed ice containing hydrochloric acid (2 N, 10 mL). The obtained solid product was filtered off, washed with water, dried, and finally recrystallized from ethanol to afford brown powder of pyridine-2,6-bis(2-cyano-*N*-phenyl-3-oxopropanethioamide) (**2**). Yield (0.41 g, 85%); mp: 114-115°C. IR (KBr pellet, cm^−1^): 3191, 2210, 1655, 1560 1468, 1380, 760, 695, 557. ^1^H NMR (300 MHz, DMSO-*d*_6_): *δ* 3.60 (s, 2H, 2CH), 7.25–7.43 (m, 10H, Ar-H), 7.74–8.02 (m, 3H, pyridine-H), 12.02 (s, 2H, 2NH, D_2_O-exchangeable). ^13^C NMR (75 MHz, DMSO-*d*_6_): *δ* 57.21, 118.70, 119.24, 123.50, 124.17, 124.95, 128.96, 129.23, 139.98, 150.21, 180.14. MS (*m/z, *%): 483 [M^+^] (17), 317 (30), 197 (14), 144 (19), 105 (55), 78 (45), 60 (100). Anal. Calcd. for C_25_H_17_N_5_S_2_O_2_ (483.56): C, 62.09; H, 3.54; N, 14.48. Found: C, 62.16; H, 3.48; N, 14.53.

#### 4.2.3. Pyridine-2,6-bis((*E*)-2-(2-phenylhydrazono)-3-oxopropanenitrile) (**3**)

To a stirred solution of pyridine-2,6-bis(3-oxopropanenitrile) (**1**) (2.13 g, 10 mmol) in EtOH (100 mL), sodium acetate trihydrate (3 g) was added. After stirring for 10 min, the mixture was cooled to −4°C and treated with aniline diazonium salt solution (prepared by diazotizing aniline (10 mmol) in hydrochloric acid (6 N, 6 mL) with sodium nitrite solution (0.7 g, 10 mmol), in 5 mL water). Addition of the diazonium salt was carried out with rapid stirring over a period of 30 min. The reaction mixture was stirred for further 2 h at −4°C and then was left for 6 h, in a refrigerator. The resulting solid was collected by filtration, washed thoroughly with water, dried, and finally recrystallized from EtOH/dioxane to give pyridine-2,6-bis((*E*)-2-(2-phenylhydrazono)-3-oxopropanenitrile) (**3**). Yield (3.5 g, 83%, yellow crystals); mp: 221–223°C. IR (KBr pellet, cm^−1^): 3187, 2213, 1674, 1549, 1478, 1430 1357, 1266, 916, 755, 683 cm^−1^. ^1^H NMR (300 MHz, DMSO-*d*_6_): *δ* 7.10–7.28 (m, 10H, ArH's), 7.97–8.20 (m, 3H, pyridine-H's) 12.63 (s, 2H, 2NH, D_2_O-exchangeable). ^13^C NMR (75 MHz, DMSO-*d*_6_): *δ* 115.11, 120.11, 125.11, 126.14, 138.05, 142.31, 150.4, 154.12, 195.17. MS* m/z *(%): 421 [M^+^] (13), 315 (46), 212 (30), 167 (27), 105 (30), 92 (25), 77 (86), 51 (100). Anal. Calcd. for C_23_H_15_N_7_O_2_ (421.41): C, 65.55; H, 3.59; N, 23.27. Found: C, 65.47; H, 3.64; N, 23.21.

### 4.3. Synthesis of 2,6-bis(substituted)pyridine Ag(I) Nitrate Complexes

#### 4.3.1. [AgL_1_(NO_3_)] (**4**)

A mixture of AgNO_3_ (0.17 g, 1 mmol) and pyridine-2,6-bis(3-oxopropanenitrile) (**1**) (0.21 g, 1 mmol) in EtOH (20 mL) was stirred at room temperature for 3 h. The precipitated product was filtered off, washed with water, dried, and finally recrystallized from EtOH/dioxane to afford yellow powder of complex** 4**.

Yield (0.27 g, 70%); IR (KBr pellet, cm^−1^): 3430, 2165, 1611, 1571, 1517, 1375, 561, 460. ^1^H NMR (300 MHz, DMSO-*d*_6_): *δ* 4.21 (s, 4H, 2CH_2_), 7.95–8.15 (m, 3H, pyridine-H). ^13^C NMR (75 MHz, DMSO-*d*_6_): *δ* 28.2, 115.4, 125.7, 126.3, 155.7, 181.7 Anal. Calcd. for C_11_H_7_AgN_4_O_5_ (387.07): C, 34.49; H, 1.84; N, 14.63. Found: C, 35.01; H, 2.01; N, 14.43.

#### 4.3.2. [AgL_2_(NO_3_)] (**5**)

A mixture of AgNO_3_ (0.17 g, 1 mmol) and pyridine-2,6-bis(2-cyano-*N*-phenyl-3-oxopropanethioamide) (**2**) in MeOH was refluxed for 4 h. The resultant solution was allowed to evaporate slowly at room temperature for several days to give yellow powder of complex** 5**. The precipitated product was filtered off, washed with water/MeOH, dried, and finally recrystallized from EtOH/DMF to afford yellow powder of complex** 5.** Yield (0.51 g, 78%); IR (KBr pellet, cm^−1^): 3386, 2198, 1621, 1553, 1473, 1321, 754, 693, 561. ^1^H NMR (300 MHz, DMSO-*d*_6_): *δ* 7.25–8.16 (m, 13H, Ar-H, pyridine-H), 12.11 (s, 2H, 2NH, D_2_O-exchangeable). ^13^C NMR (75 MHz, DMSO-*d*_6_): *δ* 116.04, 121.31, 125.23, 125.81, 138.11, 141.33, 150.4, 160.17, 185.85. Anal. Calcd for C_25_H_17_AgN_6_O_5_S_2_ (653.44): C, 45.95; H, 2.62; N, 12.86. Found: C, 46.12; H, 2.75; N, 12.45.

#### 4.3.3. [AgL_3_(NO)] (**6**)

To a stirred solution of AgNO_3_ (0.17 g, 1 mmol) in EtOH (20 mL), pyridine-2,6-bis((*E*)-2-(2-phenylhydrazono)-3-oxopropanenitrile) (**3**) (0.42 g, 1 mmol) was added. The reaction mixture was refluxed for 8 h and then allowed to cool. The formed solid product was filtered off, washed with ethanol/water, dried, and recrystallized from EtOH/dioxane to afford complex** 6**. Yield (0.41 g, 70%); IR (KBr pellet, cm^−1^): 3430 (NH), 2167 (CN), 1707 (C=O), 1512 (C=N pyridine moiety), 460 (C=O–Ag), 441 (Ag–N). ^1^H NMR (300 MHz, DMSO-*d*_6_): *δ* 7.22–7.47 (m, 10H, ArH's), 8.21–8.42 (m, 3H, pyridine-H), 12.10 (br s, 2H, 2NH, D_2_O-exchangeable). ^13^C NMR (75 MHz, DMSO-*d*_6_): *δ* 57.21, 117.15, 122.25, 125.50, 126.17, 127.95, 128.86, 129.52, 138.45, 150.52, 194.14.Anal. Calcd for C_23_H_15_AgN_8_O_5_ (591.28): C, 46.72; H, 2.56; N, 18.95. Found: C, 46.52; H, 2.36; N, 18.65.

### 4.4. *In Vitro* Assay for Anticancer Activity

The synthesized compounds were supplied to the Bioassay-Cell Culture Laboratory, National Research Centre, Cairo, Egypt, for* in vitro *antitumor screening on Lung adenocarcinoma (A549), hepatocellular carcinoma (HePG2), colon carcinoma (HT29), and caucasian breast adenocarcinoma (MCF7) (American Type Culture Collection). Cell viability was assessed by the mitochondrial-dependent reduction of yellow MTT (3-(4,5-dimethylthiazol-2-yl)-2,5-diphenyl tetrazolium bromide) to purple formazan [34, 35].

*Procedure.* All the following procedures were done in a sterile area using a Laminar flow cabinet biosafety class II level (Baker, SG403INT, Sanford, ME, USA). HePG2 cell line was cultured in RPMI-1640, and MCF7 cell line was cultured in DMEM. Cells were plated in 96-well plates (having about 10000 cells/well). The plates are then incubated for 24 h in 37°C incubation and 5% CO_2_ atmosphere before treatment with the compounds to allow attachment of cell to the wall of the plate. Tested compounds were dissolved in DMSO and different concentrations of the compounds under test were added to the cell monolayer. Triplicate wells were prepared for each individual concentration. Then the plate was incubated for 48 h in 37°C incubator. After the completion of compound exposure, 40 *μ*L of MTT solution (2.5 mg/mL) was added into each well for an additional 4 h. Formazan was dissolved in 200 *μ*L (10%) sodium dodecyl sulphate, and the absorbance at *λ* = 495 nm was measured. The concentration of DMSO as a solvent for the different compounds was 0.1% in the culture medium used and was without any effect on cell growth. Cell viability at given compound concentration was calculated as the percentage of absorbance in wells with the compound-treated cells to that of vehicle control cells (100%).

## Figures and Tables

**Table 1:** Anticancer screening of the newly synthesized ligands and Ag(I) nitrate complexes against the tested human cancer cell lines.

Compound	Cytotoxicity^a,b^ (IC_50_) (*μ*M)			
	HEPG2	A549	HT29	MCF7
**1**	45.11	NA	NA	NA
**2**	NA	NA	NA	NA
**3**	NA	34.55	49.82	29.5
**4**	1.52	1.41	1.75	3.67
**5**	6.12	1.08	8.85	10.72
**6**	1.62	3.71	52.65	3.66
Doxorubicin	4.61	3.51	5.22	2.78

^a^IC_50_, compound concentration required to inhibit tumor cell proliferation by 50%.

^b^The same experiment is carried out in triplicate.

Sample concentration range (100—0.78 *μ*M) using MTT assay.

NA: compounds having IC_50_ more than 60 *μ*M.
